# Trehalose improves the movement ability of Aβ_arc_ *Drosophila* by restoring the damaged mitochondria

**DOI:** 10.1515/tnsci-2022-0338

**Published:** 2024-04-10

**Authors:** Liangxian Li, Zhiheng Huang, Mingli Wu, Xia Li, Bo Xiao, Dong Yao, Biwen Mo

**Affiliations:** Department of Respiratory and Critical Care Medicine, The Second Affiliated Hospital of Guilin Medical University, Guilin, 541199, China; Guangxi Key Laboratory of Brain and Cognitive Neuroscience, Guilin Medical University, Guilin, 541199, China; Laboratory of Respiratory Disease, Affiliated Hospital of Guilin Medical University, Guilin, 541002, China; Laboratory of Basic Research on Respiratory Diseases, Guangxi Health Commission, Guilin Medical University, Guilin, 541199, China; Guangxi Clinical Research Center for Diabetes and Metabolic Diseases, Guangxi Health Commission Key Laboratory of Glucose and Lipid Metabolism Disorders, The Second Affiliated Hospital of Guilin Medical University, 541199, Guilin, China; Guangxi Key Laboratory of Metabolic Reprogramming and Intelligent Medical Engineering for Chronic Diseases, The Key Laboratory of Respiratory Diseases, Education Department of Guangxi Zhuang Autonomous Region, Guilin Medical University, 541199, Guilin, China

**Keywords:** Alzheimer’s disease, Aβ_arc_ *Drosophila*, trehalose, mitochondria, movement ability

## Abstract

**Background:**

The deposition of Aβ_42_ has been regarded as one of the important pathological features of Alzheimer’s disease (AD). However, drug development for Aβ_42_ toxicity has been progressed slowly.

**Objective:**

Our aim was to introduce the effect and related mechanism of trehalose on an Aβ_arc_ (arctic mutant Aβ_42_) *Drosophila* AD model.

**Methods:**

The human Aβ_arc_ was expressed in *Drosophila* to construct the AD model. Trehalose was added to the culture vial. The movement ability was determined by detecting climbing ability and flight ability. Enzyme-linked immunosorbent assay was used to detect the levels of Aβ_arc_, ATP, and lactate. Electron microscopy assay, mitochondrial membrane potential assay, and mitochondrial respiration assay were used to assess the mitochondrial structure and function.

**Results:**

Trehalose strongly improved the movement ability of Aβ_arc_ *Drosophila* in a concentration gradient-dependent manner. Furthermore, trehalose increased the content of ATP and decreased the content of Aβ_arc_ and lactate both in the brain and thorax of Aβ_arc_ *Drosophila*. More importantly, the mitochondrial structure and function were greatly improved by trehalose treatment in Aβ_arc_ *Drosophila*.

**Conclusion:**

Trehalose improves movement ability at least partly by reducing the Aβ_arc_ level and restoring the mitochondrial structure and function in Aβ_arc_ *Drosophila*.

## 1 Introduction

Alzheimer’s disease (AD) is the most common form of senile dementia [1]. The accumulation and deposition of β amyloid (Aβ) is one of the most important pathological features of AD [2]. Recently, a widely accepted hypothesis suggests that the main pathological features in AD, including Tau protein hyperphosphorylation, glial cell activation, inflammation, synaptic damage, oxidative stress, and energy metabolism damage, are all attributed to the accumulation of soluble Aβ [3]. Therefore, developing drugs targeting Aβ would be useful for anti-AD.

Due to its small size, easy reproduction, and ease of genetic manipulation, *Drosophila* has been widely used to construct AD models [4]. *Drosophila* AD models were almost constructed by expressing human Aβ, APP, BACE, and presenilin [5]. These AD models could effectively imitate the pathological state of AD patients [5]. Arctic mutant Aβ_42_ (Aβ_arc_) is an important mutation of Aβ_42_ in familial Alzheimer’s disease [6]. The expression of Aβ_arc_ exhibited stronger neurotoxicity than the expression of Aβ_40_ and Aβ_42_ in *Drosophila* [7,8]. In other words, Aβ_arc_ *Drosophila* AD model is more suitable to explore drugs for AD.

Trehalose is a safe and reliable natural non-reducing disaccharide, widely present in non-mammalian animals, plants, and microorganisms, with excellent capabilities of anti-inflammatory and antioxidant stress [9]. Currently, trehalose has been reported in various neurodegenerative diseases, mainly by activating autophagy to improve related pathological features [10]. In AD, most studies have focused on evaluating the effects of trehalose on AD cell models *in vitro* [11,12,13,14,15], which makes the progress of trehalose as a therapeutic drug for AD very slow. Only several studies simply reported that trehalose could improve cognition in several types of transgenic mice, such as Tg2576, APP23, and APP/PS1 mice [16,17,18]. It has still been unclear whether trehalose could reduce the toxicity of Aβ and its mechanism *in vivo*.

In this study, we first reported that trehalose strongly improves the climbing ability and flight ability of Aβ_arc_ *Drosophila*. We also found that trehalose significantly reduces the content of Aβ_arc_ both in the brain and thorax of Aβ_arc_ *Drosophila*. Energy is directly linked to the movement ability of Aβ_arc_ *Drosophila* [19]. Therefore, we detected the levels of ATP and lactate both in the brain and thorax of Aβ_arc_ *Drosophila*. The results showed that trehalose significantly increases the ATP level and decreases the lactate level both in the brain and thorax of Aβ_arc_ *Drosophila*. Mitochondria are the main organelles for ATP production [20]. We further detected the structure and function of mitochondria. Excitedly, the results showed that trehalose greatly restores the mitochondrial structure and function damaged by Aβ_arc_ toxicity. These results indicated that Aβ_arc_ consumption and mitochondrial repairment may be the key mechanism for trehalose to rescue Aβ_arc_ *Drosophila*.

In summary, our study implied that trehalose could greatly improve the movement ability of Aβ_arc_ *Drosophila*. This improvement was realized at least partially through reducing the content of Aβ_arc_ and restoring the damaged mitochondria in Aβ_arc_ *Drosophila*. In other words, trehalose could be a potential therapeutic drug for the treatment of AD.

## 2 Materials and methods

### 2.1 *Drosophila* stocks

The cultured conditions for *Drosophila* stocks were described as follows. First, the culture medium is composed of multiple components, such as ddH_2_O 0.65 L/L, yeast 15 g/L, corn flour 38.85 g/L, sucrose 15.81 g/L, glucose 31.6 g/L, methyl *p*-hydroxybenzoate 0.75 g/L (soluble in alcohol 7.5 mL), and agar 5.6 g/L. Second, *Drosophila* was placed into an incubator with 12 h/12 h light/dark cycle, 25°C, and 50–70% relative humidity. W^1118^ *Drosophila* (Bloomington stock, #5905) was obtained from *Drosophila* Bloomington Stock Center (University of Indiana, Bloomington, IN). The upstream activating sequence transgenic line used for expressing Aβ_arc_ (P{UAS-Aβ_arc_}) and [Gal4]A307 transgenic line used for driving the expression of Aβ_arc_ in giant fiber (GF) system and other components of the nervous system were generous gifts from Dr. Fu-De Huang (Institute of Neuroscience and State Key Laboratory of Neuroscience, Shanghai, China) [7]. P{UAS-Aβ_arc_} was crossed to [GAL4]A307 (virgin flies) line to generate heterozygous flies expressing Aβ_arc_. [Gal4]A307 (virgin flies) was crossed to W^1118^ to generate control flies containing one copy of [Gal4]A307. Before use, all these transgenic flies were separately backcrossed to an isogenic control line W^1118^ for at least five generations. Male flies were collected and used in this study.

### 2.2 Drug intervention

Trehalose (Yuanye, Shanghai, China, # S11051) was dissolved and diluted to the final concentrations with ddH_2_O in this study. The administration method was completely based on a previous study [19]. Briefly, 80 μl of trehalose solution with the final concentrations (0, 50, 100, and 200 mM) was daily added to the culture vial (with sufficient food) containing 20 newly eclosed subject flies (1–2 days old) until 25 days. The flies were transferred into fresh food every 7 days. The groups were designed as follows: wild-type group: A307  >  W^1118^, Aβ_arc_ expression group: A307  >  Aβarc, Aβ_arc_ expression plus low-dose trehalose treatment group (TRE50): A307  >  Aβarc +  TRE 50 mM, Aβ_arc_ expression plus middle-dose trehalose treatment group (TRE100): A307  >  Aβarc +  TRE 100 mM, and Aβ_arc_ expression plus high-dose trehalose treatment group (TRE200): A307  >  Aβarc +  TRE 200 mM.

### 2.3 Climbing assay

The climbing assay was performed according to the negative geotaxis climbing assay as previously described [19]. Briefly, flies (*n* = 30, 25 days after eclosion) were placed into a transparent testing vial (a diameter of 2.1 cm and height of 19.0 cm) and tapped down to the bottom of the vial. So the flies were allowed to climb upwards the walls of the vial due to negative geotaxis. A digital video recorder was used to record the climbing process. The flies were appraised in 3–5 consecutive trials separated by 40 s intervals. The height of each fly in each vial at 20 s was measured by software “RflyDetection” to evaluate the fly’s climbing ability. All behavioral recording was done at 25°C.

### 2.4 Flight ability

The flight ability detection was performed as the previous study described [19]. Briefly, the single fly was tapped down into a glass cylinder (inner diameter of 10 cm and length of 39 cm), which was divided into 13 zones of 3 cm each. The zone in which the fly landed was recorded and used to evaluate the landing height. Ten flies were used in each group.

### 2.5 Detection of Aβ_arc_, ATP, and lactate

Brains and thoraces of the flies (*n* = 50, 25 days after eclosion) in each group were homogenized (Tissue homogenizer, Next Advance) thoroughly in cold RIPA buffer (Solarbio, # R0020) supplemented with cocktail protease inhibitor (bimake, # B14001). The samples were incubated on ice for 30 min and then centrifuged at 12,000*g* for 10 min at 4°C. Supernatants were collected for enzyme-linked immunosorbent assay (ELISA) analysis. The content of Aβ_arc_ (Invitrogen, # KHB3441), ATP (Beyotime, # S0026), and lactate (Sigma-Aldrich, MAK064) was determined according to the manufacturer’s instructions respectively.

### 2.6 Immunostaining

Brains of the flies were dissected in pre-cooling PBS and fixed with 4% paraformaldehyde for 1 h, washed with 0.3% Triton X-100 for 5 min (five times), and then treated with 70% formic acid for 45 min to re-expose the epitope. The brains were then washed with 0.3% Triton X-100 for 5 min (three times) and blocked with 5% normal goat serum (Solarbio, # SL038) at room temperature for 30 min. The brains were then incubated with primary antibody (Beta Amyloid, 1:100, Covance, # SIG-39300) overnight at 4°C, washed with 0.3% Triton X-100 for 10 min (five times), and then incubated with FITC-conjugated secondary goat anti-mouse antibody (ZSGB-BIO, 1:100, # ZF-0312) at room temperature for 2 h in dark. A laser scanning confocal microscope (Olympus FV3000) at the step of 1 μm was used to acquire the projection of Z-stack images. The standard images of Aβ were taken.

### 2.7 Quantitative real-time PCR (qRT-PCR)

Brains of the flies (*n* = 20, 25 days after eclosion) in each group were homogenized thoroughly with 1 mL of TRI reagent (MRC, # TR118). Total RNAs were extracted according to the manufacturer’s instructions. cDNAs were obtained with PrimeScript™ RT Reagent Kit with gDNA Eraser (Takara, # RR047A). qRT-PCR was performed with MonAmp™ SYBR^®^ Green qPCR Mix (Monad, # MQ10101S) according to the manufacturer’s instructions. The primers were described as follows: forward primer of Aβ_arc_: ATGGCGAGCAAAGTCTCGATC, reverse primer of Aβ_arc_: CGCAATCACCACGCCGCCCAC; forward primer of 18S: TCTAGCAATATGAGATTGAGCAATAAG, reverse primer of 18S: AATACACGTTGATACTTTCATTGTAGC. The expression level of Aβ_arc_ was normalized to 18S.

### 2.8 Electron microscopy

Thoraces of the flies were dissected and fixed in 2.5% glutaraldehyde and 1% osmium tetroxide and embedded in Epon resin as previously described (standard procedures optimized for *Drosophila* tissue) [19]. Ultra-thin sections were stained with uranyl acetate and lead citrate. HT7700 TEM (HITACHI, Japan) was used for imaging. Broken mitochondria were defined as previously described [21]. Broken mitochondria were quantified in each group by manual counting.

### 2.9 Mitochondrial respiration assay

Oxygraph-2 K high-resolution respirometry (Oxygraph-2K 10000-1, Oroboros, AT) was used to detect mitochondrial respiration. Brains of the flies (*n* = 10, 25 days after eclosion) in each group were homogenized thoroughly on ice using a pestle in MiR05 respiration buffer (10 mM KH_2_PO_4_, 20 mM HEPES, 110 mM sucrose, 60 mM K-lactobionate, 20 mM taurine, 0.5 mM EGTA, 3 mM MgCl_2_, and 1 g/l fatty acid-free BSA). Substrates, uncoupler, and inhibitors of mitochondrial respiratory chain complexes were used as follows: substrates including 2 M pyruvate, 0.8 M malate, 2 M glutamate, 1 M succinate, 0.5 M ADP + Mg^2+^, and 4 mM cytochrome C; uncoupler including 1 mM carbonyl cyanide m-chlorophenyl hydrazine; and inhibitors including 1 mM rotenone and 5 mM Antimycin A. Complex І respiration was measured in MiR05 respiration buffer in the presence of pyruvate, malate, glutamate, and ADP + Mg^2+^. Complex II was assayed in respiration buffer supplemented with rotenone and succinate. Oxygen concentration and oxygen flux indicating the function of complex І and complex Ⅱ were recorded using DatLab software (Oroboros Instruments) as previously described [19].

### 2.10 Determination of mitochondrial membrane potential

Brains of the flies (*n* = 50, 25 days after eclosion) in each group were used to extract mitochondria with the Tissue Mitochondria Isolation Kit (Beyotime, # C3606). Mitochondrial membrane potential was determined by Enhanced Mitochondrial Membrane Potential Assay Kit with JC-1 (Beyotime, # C2003S) according to the manufacturer’s instructions.

### 2.11 Statistical analysis

Data analysis was conducted by GraphPad Prism software. Multiple groups’ comparison used one-way analysis of variance (ANOVA) followed by Tukey’s post hoc test. Two groups’ comparison used a two-tailed unpaired Student’s *t* test. Significance was considered at *P ≤ 0.05, **P ≤ 0.01, ***P ≤ 0.005, or ****P ≤ 0.001 in this study. The data were represented as mean ± SD. All experiments were run at least in triplicate.

## 3 Results

### 3.1 The construction of Aβ_arc_ *Drosophila* AD model

**Figure 1:**
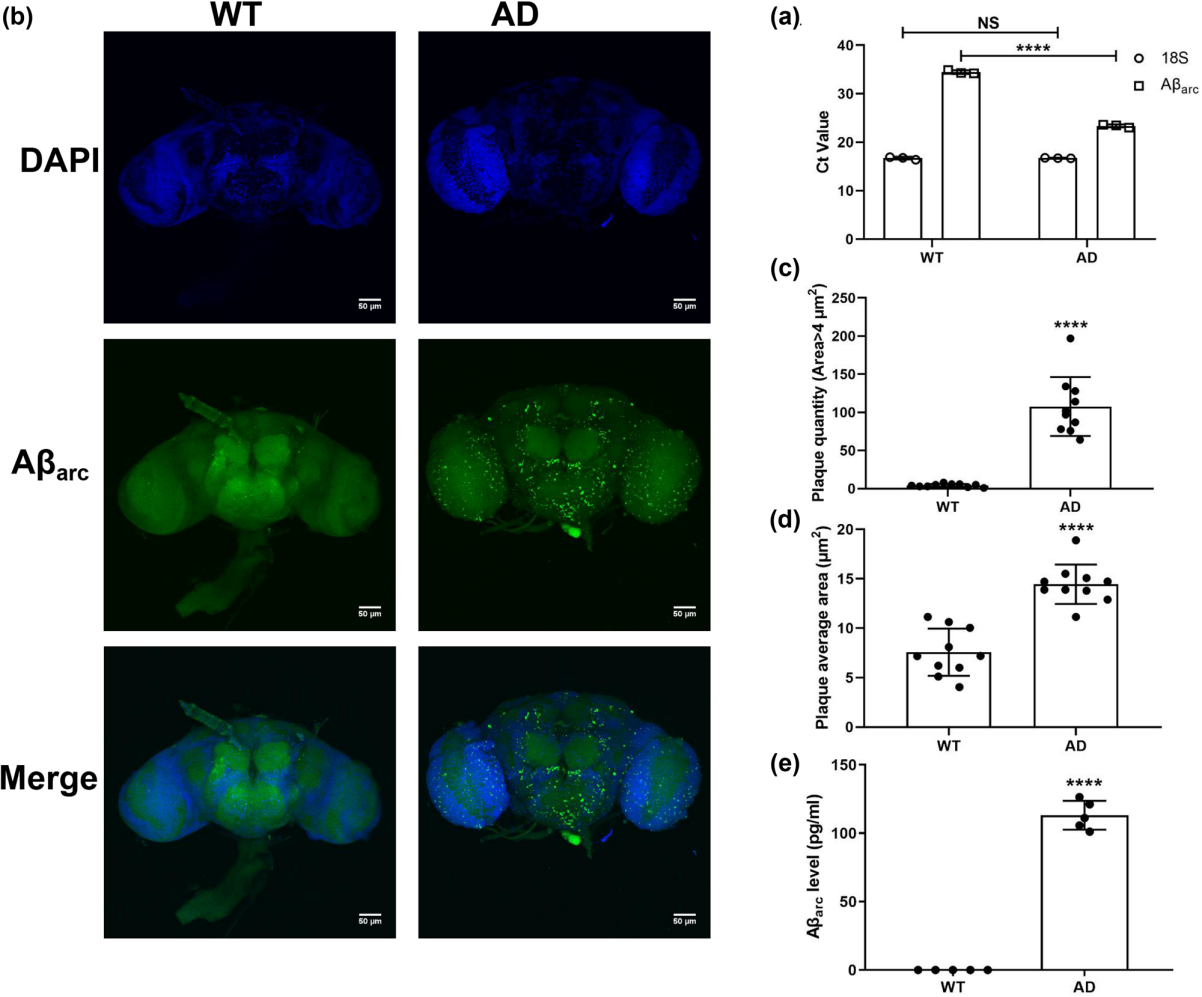
The construction of Aβ_arc_ *Drosophila* AD model. (a) Determining the Ct value of 18S and Aβ_arc_ by qRT-PCR. (b) Immunostaining of Aβ_arc_ (whole brain; *n* = 10). (c) Quantitative analysis of plaque quantity (staining with Aβ_arc_) in (b). (d) Quantitative analysis of plaque average area (staining with Aβ_arc_) in (b). (e) Determining the content of soluble Aβ_arc_. WT: wild-type group; AD: Aβ_arc_ expression group. Error bars represent the SD of at least three independent experiments. NS represents not significant. Scale bars, 50 μm. ****P ≤ 0.001 use an unpaired two-tailed Student’s *t*-test.

Aβ_arc_ *Drosophila* was used as an AD model in this study. The qRT-PCR results showed that the gene expression of Aβ_arc_ was confirmed in Aβ_arc_ *Drosophila* (Figure 1a). The immunostaining also showed that the deposition of Aβ_arc_ exists in the brain of Aβ_arc_ *Drosophila* (Figure 1b). The analysis of Figure 1b found that both the plaque quantity (area >4 μm^2^) and the plaque average area significantly increased in Aβ_arc_ *Drosophila* (Figure 1c and d). The ELISA also found that soluble Aβ_arc_ significantly increases in Aβ_arc_ *Drosophila* (Figure 1e).

### 3.2 Trehalose improves the movement ability of Aβ_arc_ *Drosophila*

**Figure 2:**
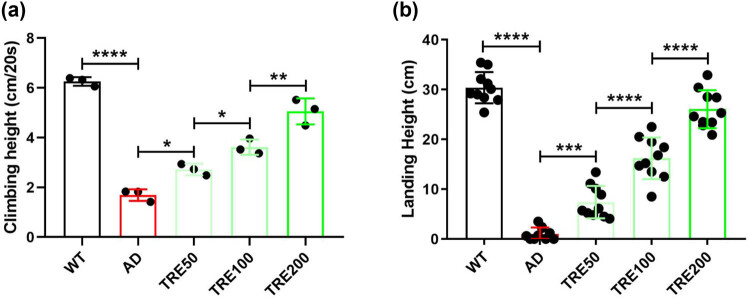
Trehalose restores the climbing ability and flight ability of Aβ_arc_ *Drosophila*. Determination of the climbing ability (*n* = 30 in each vial) and flight ability (*n* = 10) in each group and the concentrations of trehalose used are 50, 100, and 200 mM. Determination of (a) climbing ability (*n* = 30 in each vial) and (b) flight ability (*n* = 10). WT: wild-type group; AD: Aβ_arc_ expression group; TRE50: Aβ_arc_ expression plus trehalose 50 mM treatment group; TRE100: Aβ_arc_ expression plus trehalose 100 mM treatment group; TRE200: Aβ_arc_ expression plus trehalose 200 mM treatment group. Error bars represent the SD of at least three independent experiments. *P ≤ 0.05, **P ≤ 0.01, ***P ≤ 0.005, and ****P ≤ 0.001 use a one-way ANOVA followed by Tukey’s post hoc test.

The climbing ability and the flight ability are always used to evaluate the movement ability of Aβ_arc_ *Drosophila*. We found that trehalose rescues the climbing ability of Aβ_arc_ *Drosophila* in a concentration gradient-dependent manner (Figure 2a). We also found that trehalose rescues the flight ability of Aβ_arc_ *Drosophila* in the same concentration gradient-dependent manner (Figure 2b). These results indicated that trehalose successfully rescues the behaviors of Aβ_arc_ *Drosophila*.

### 3.3 Trehalose reduces the content of Aβ_arc_ in Aβ_arc_ *Drosophila*

**Figure 3:**
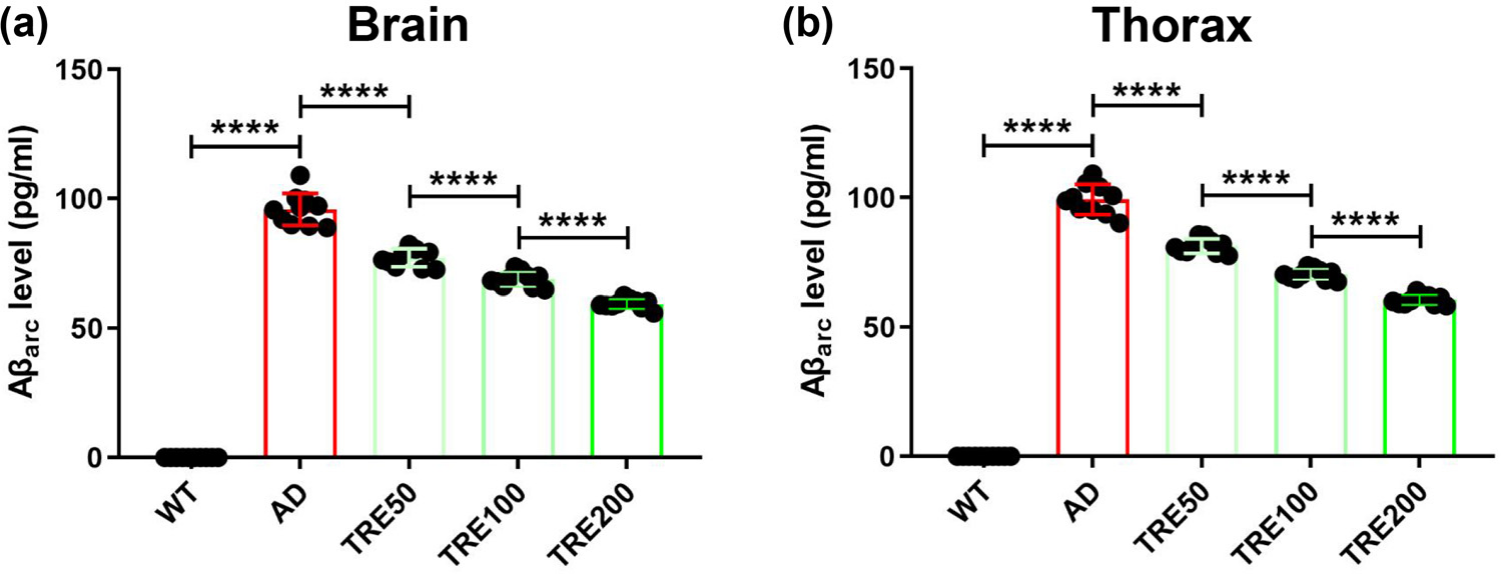
Trehalose reduces the content of Aβ_arc_ in Aβ_arc_ *Drosophila*. (a) Measuring the content of Aβ_arc_ in the brain. (b) Determining the content of Aβ_arc_ in the thorax. WT: wild-type group; AD: Aβ_arc_ expression group; TRE50: Aβ_arc_ expression plus trehalose 50 mM treatment group; TRE100: Aβ_arc_ expression plus trehalose 100 mM treatment group; TRE200: Aβ_arc_ expression plus trehalose 200 mM treatment group. Error bars represent the SD of at ten independent experiments. ****P ≤ 0.001 use a one-way ANOVA followed by Tukey’s post hoc test.

Aβ_arc_ toxicity is directly related to the content of Aβ_arc_. We detected the level of Aβ_arc_ in Aβ_arc_ *Drosophila*. We found that trehalose reduces the content of Aβ_arc_ both in the brain and thorax of Aβ_arc_ *Drosophila* with a concentration gradient-dependent manner (Figure 3a and b). These results indicated that trehalose could significantly reduce the Aβ_arc_ toxicity.

### 3.4 Trehalose significantly restores the ATP and lactate levels

**Figure 4:**
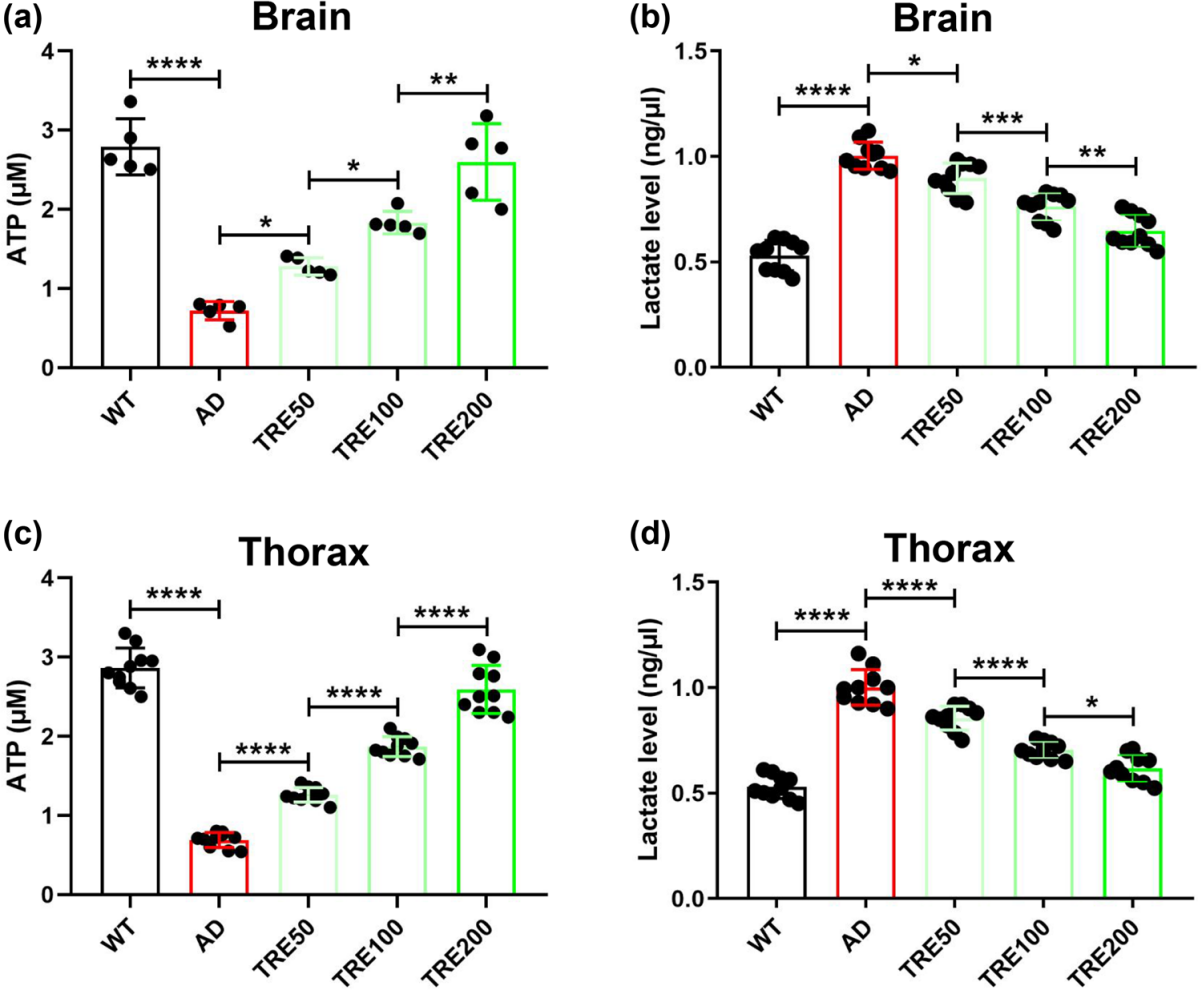
Trehalose rescues the production of ATP and lactate in Aβ_arc_ *Drosophila*. (a) Measuring the content of ATP in the brain. (b) Determining the content of lactate in the brain. (c) Measuring the content of ATP in the thorax. (d) Determining the content of lactate in the thorax. WT: wild-type group; AD: Aβ_arc_ expression group; TRE50: Aβ_arc_ expression plus trehalose 50 mM treatment group; TRE100: Aβ_arc_ expression plus trehalose 100 mM treatment group; TRE200: Aβ_arc_ expression plus trehalose 200 mM treatment group. Error bars represent the SD of at least five independent experiments. *P ≤ 0.05, **P ≤ 0.01, ***P ≤ 0.005, and ****P ≤ 0.001 use a one-way ANOVA followed by Tukey’s post hoc test.

The improvement of energy metabolism is an important prerequisite for enhancing movement ability. We found that trehalose greatly increases the ATP production both in the brain and thorax of Aβ_arc_ *Drosophila* (Figure 4a and b). The improvements exhibited a gradient dependence of trehalose concentration (Figure 4a and b). We also found that trehalose attenuates the accumulation of lactate both in the brain and thorax of Aβ_arc_ *Drosophila* in the same concentration-dependent manner (Figure 4c and d). These results demonstrated that trehalose could enhance the movement ability of Aβ_arc_ *Drosophila* by improving damaged energy metabolism.

### 3.5 Trehalose strongly restores the damaged mitochondria

**Figure 5:**
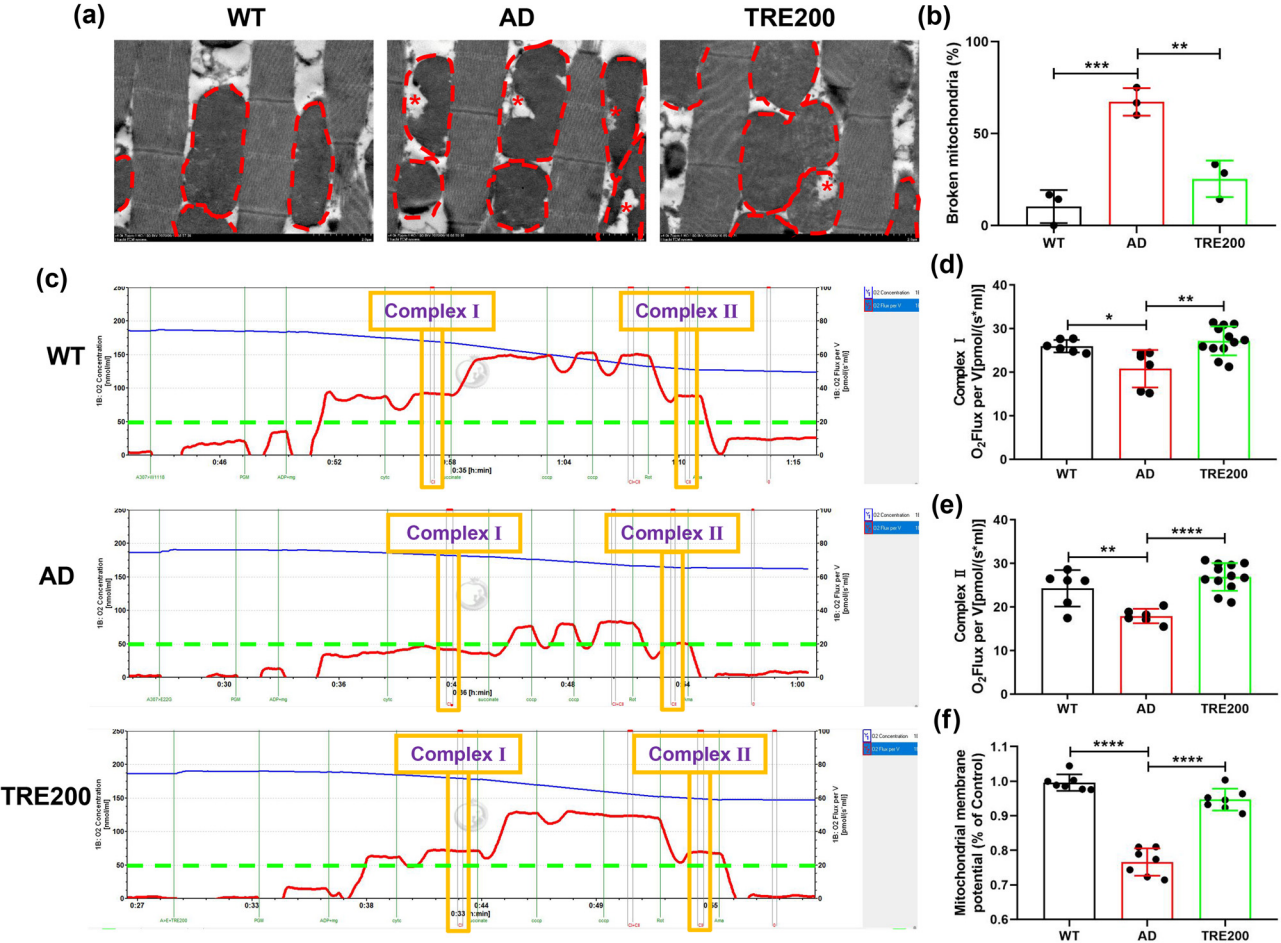
Trehalose restores the damaged mitochondria in Aβ_arc_ *Drosophila*. (a) The red dashed line represents mitochondria in each group, and the red asterisk represents the location of mitochondrial structural disruption. (b) Determining the number of mitochondria without intact structure in (a). (c) Mitochondrial respiration in each group. (d) Analyzing the Complex І respiration in (c). (e) Analyzing the Complex Ⅱ respiration in (c). (f) Determining the mitochondrial membrane potential. WT: wild-type group; AD: Aβ_arc_ expression group; TRE200: Aβ_arc_ expression plus trehalose 200 mM treatment group. Error bars represent the SD of at least three independent experiments. *P ≤ 0.05, **P ≤ 0.01, ***P ≤ 0.005, and ****P ≤ 0.001 use a one-way ANOVA followed by Tukey’s post hoc test. Scale bars, 2 μm.

Mitochondria are the most important organelles for ATP production. Electron microscopy analysis showed that trehalose restores the mitochondrial structure damaged by Aβ_arc_ toxicity (Figure 5a and b). We also found that trehalose greatly ameliorates the mitochondrial function damaged by Aβ_arc_ toxicity (Figure 5c). In detail, mitochondria functional analysis showed that trehalose dramatically restores the function of complex Ⅰ and complex Ⅱ disrupted by Aβ_arc_ toxicity (Figure 5d and e). Moreover, mitochondrial membrane potential analysis showed that trehalose could repair the mitochondrial structure damaged in Aβ_arc_ *Drosophila* (Figure 5f). The above results indicated that mitochondria may be the targeted organelles for trehalose to rescue Aβ_arc_ *Drosophila*.

## 4 Discussion

Aβ has always played a very important role in the definition of pathological features in AD [1]. It is a consensus that the accumulation and deposition of Aβ is the main cause of the cliff-like decline in motor function in AD patients [3]. Therefore, the development of drugs targeting the toxicity of Aβ is crucial.

Trehalose has played a therapeutic role in various neurodegenerative diseases, especially amyotrophic lateral sclerosis [10,22,23,24,25,26]. However, the effects of trehalose on AD mostly focused on AD cell models just *in vitro*. Liu et al. found that trehalose differentially inhibits aggregation and neurotoxicity of Aβ_40_ and Aβ_42_ in human neuroblastoma cells (SH-SY5Y) [11]. Reddy et al. found that trehalose promotes the insertion of α-helical Aβ into biological membranes *in vitro* [12]. Krüger et al. found that trehalose suppresses Tau aggregation by activating autophagy in mouse neuroblastoma cell line N2a [13]. Tien et al. found that trehalose decreases the lysosomal metabolism of APP by altering its endocytic vesicular transport in SH-SY5Y [14]. Benito-Cuesta et al. found that the neuroprotective effect of trehalose is mediated by a reduced colocalization of APP and BACE1 in primary neurons [15]. There has not been clear enough whether trehalose could exert the expected anti-AD effects *in vivo*. Only several studies simply reported that trehalose could improve cognition in several types of transgenic mice, such as Tg2576, APP23, and APP/PS1 mice [16,17,18]. There still has been no study to evaluate the effect of trehalose on Aβ toxicity and its mechanism *in vivo*. We found for the first time that trehalose significantly improves the movement ability damaged by Aβ toxicity in Aβ_arc_ *Drosophila*. This has taken a big step forward in its treatment of AD, greatly improving our understanding of trehalose therapy.

The damage of energy metabolism has been widely recognized in AD [27]. ATP is the main carrier of energy for the body [28]. The declined ATP production is closely related to the damaged movement ability in AD [29]. Excitingly, we investigated that trehalose significantly elevates the ATP levels both in the brain and thorax of Aβ_arc_ *Drosophila*. Similarly, the accumulation of lactate is always a hallmark of energy metabolism damage [30]. We also found that trehalose decreases the lactate levels both in the brain and thorax of Aβ_arc_ *Drosophila*. These results implied that trehalose could improve energy metabolism to combat the damaged movement ability of AD *in vivo*.

ATP is mainly produced by mitochondria [31]. Previous studies showed that therapeutic drugs and strategies targeting mitochondria can effectively rescue AD [32]. And the strength of AD resistance is closely related to the supply of ATP [33]. Therefore, we focused on the effects of trehalose on the damaged mitochondria in Aβ_arc_ *Drosophila*. Mitochondrial function analysis found that trehalose effectively repairs the damaged mitochondrial function in Aβ_arc_ *Drosophila*. And mitochondrial structure analysis also found that trehalose effectively repairs the damaged mitochondrial structure in Aβ_arc_ *Drosophila*. These results suggested that mitochondria should be the targeted organelles of trehalose for anti-AD.

Moreover, mitochondrial cascade hypothesis proposes that mitochondrial dysfunction drive the pathogenesis of AD [34]. Toxin-induced mitochondrial dysfunction drives Aβ production [32]. Aβ also drives mitochondrial dysfunction [32]. It means that mitochondrial dysfunction and Aβ production may form a vicious cycle, leading to a rapid deterioration of AD. We also found that trehalose reduces the level of Aβ_arc_ in Aβ_arc_ *Drosophila*. It implied that trehalose may be a candidate drug to break this vicious cycle. Otherwise, mitochondria can directly uptake Aβ via the TOM import machinery [35]. Subsequently, the mitochondrial peptidase, named PreP peptidasome, can degrade the Aβ, which is uptake by mitochondria [36]. This may be the reason for the decrease in Aβ_arc_.

In addition, since Aβ tends to bind to a wide range of molecules, trehalose may directly interact with Aβ_arc_ thereby preventing the formation of toxic Aβ_arc_. In future, this is a very interesting research direction for further exploring the mechanism of trehalose against Aβ toxicity.

## 5 Conclusions

**Figure 6:**
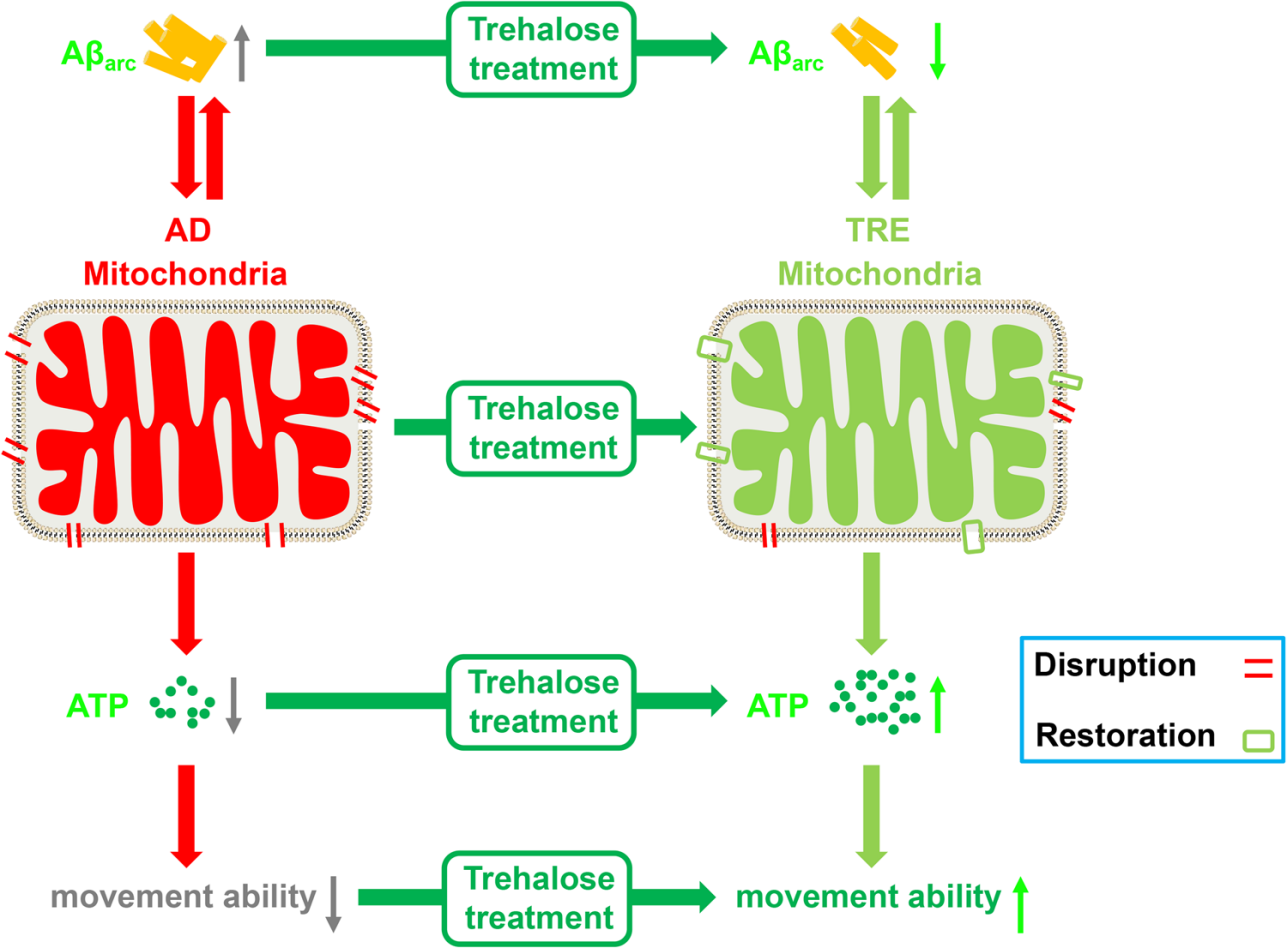
Schematic diagram of the mechanism of trehalose against Aβ_arc_ toxicity. The red parallel lines represent that Aβ_arc_ disrupts the mitochondria. The light green rectangles represent that trehalose restores the disruptions by Aβ_arc_ toxicity. AD: Aβ_arc_ expression group; TRE: Aβ_arc_ expression plus trehalose treatment group.

In summary, our results suggested that Aβ_arc_-mitochondria-ATP-movement ability is a potential axis for trehalose to rescue Aβ_arc_ *Drosophila* AD model. In other words, trehalose could improve the movement ability of AD at least partially through the Aβ_arc_-mitochondria-ATP-movement ability signal axis (Figure 6).
